# Microscale Diffusion Measurements and Simulation of a Scaffold with a Permeable Strut

**DOI:** 10.3390/ijms141020157

**Published:** 2013-10-10

**Authors:** Seung Youl Lee, Byung Ryong Lee, Jongwan Lee, Seongjun Kim, Jung Kyung Kim, Young Hun Jeong, Songwan Jin

**Affiliations:** 1 Department of Mechanical System Engineering, Graduate School of Knowledge-Based Technology and Energy, Korea Polytechnic University, Jeongwang-dong, Siheung-si, Gyeonggi-do 429-793, Korea; E-Mails: leelsyy@naver.com (S.Y.L.); jw0295@gmail.com (J.L.); 2 Department of Mechanical Engineering, Korea Polytechnic University, Jeongwang-dong, Siheung-si, Gyeonggi-do 429-793, Korea; E-Mails: conzoid25@gmail.com (B.R.L.); yhjeong@kpu.ac.kr (Y.H.J.); 3 Department of Mechanical Engineering, Graduate School, Kookmin University, Jeongneung-ro 77, Seongbuk-gu, Seoul 136-702, Korea; E-Mail: qkekdidk@hanmail.net; 4 School of Mechanical Systems Engineering, Kookmin University, Jeongneung-ro 77, Seongbuk-gu, Seoul 136-702, Korea; E-Mail: jkkim@kookmin.ac.kr

**Keywords:** diffusion, electrospinning, fluorescence recovery after photobleaching, scaffold, nanofiber

## Abstract

Electrospun nanofibrous structures provide good performance to scaffolds in tissue engineering. We measured the local diffusion coefficients of 3-kDa FITC-dextran in line patterns of electrospun nanofibrous structures fabricated by the direct-write electrospinning (DWES) technique using the fluorescence recovery after photobleaching (FRAP) method. No significant differences were detected between DWES line patterns fabricated with polymer supplied at flow rates of 0.1 and 0.5 mL/h. The oxygen diffusion coefficients of samples were estimated to be ~92%–94% of the oxygen diffusion coefficient in water based on the measured diffusion coefficient of 3-kDa FITC-dextran. We also simulated cell growth and distribution within spatially patterned scaffolds with struts consisting of either oxygen-permeable or non-permeable material. The permeable strut scaffolds exhibited enhanced cell growth. Saturated depths at which cells could grow to confluence were 15% deeper for the permeable strut scaffolds than for the non-permeable strut scaffold.

## 1. Introduction

The goal of tissue engineering is tissue regeneration. Tissue-engineering scaffolds provide the three-dimensional (3D) space necessary for cells to grow into tissues and to then guide their development into specific shapes [1,2]. Various scaffold-fabrication methods are available, such as emulsion freeze-drying [3,4], foaming [5,6], salt leaching [7], rapid prototyping [8–11], and electrospinning [12–14]. Various synthetic polymers, such as polystyrene (PS) [15], poly(lactic-co-glycolic acid) (PLGA) [9,10,16] and polycaprolactone (PCL) [9–11,16], as well as natural polymers, such as chitosan [17–23] and silk [24–26], have been used as scaffold materials.

Electrospun nanofibers have high surface-to-volume ratios and a structure similar to that of the extracellular matrices of the human body. Electrospinning techniques use a variety of materials, including natural materials such as collagen and alginate. These characteristics enable good performance in terms of cell adhesion; therefore, a number of recent studies have investigated electrospun scaffolds [12–14]. However, difficulties related to structural control have been identified as a drawback of electrospinning. Therefore, most electrospinning products are two-dimensional (2D) sheets, unlike products fabricated using rapid prototyping. We recently reported a direct-write electrospinning (DWES) technique for patterning electrospun nanofibers [27]. Various patterns, such as lines, spirals, and meshes were successfully fabricated using this technique. The outward shape of the patterns resembles those fabricated using rapid prototyping, but each pattern is composed of tangled nanofibers with improved consistency in terms of geometry and morphology. Therefore, scaffolds fabricated using the DWES technique have the advantages of both electrospun nanofibers and rapid prototyping simultaneously.

The permeability of struts is another advantage of patterned electrospun scaffolds. The permeability of a “scaffold” is determined by the arrangement and volume fraction of struts, whereas the permeability of the “struts” is determined by the properties of the strut material. A scaffold with permeable struts is expected to improve mass transfer to the inner regions of the scaffold, especially when cells fill the spaces between the struts. Limited mass transfer into the scaffold interior is directly linked to necrosis of internally located cells, a phenomenon observed in most scaffolds developed to date. Necrosis typically occurs when oxygen cannot diffuse further into the interior of a scaffold where cells proliferate [28]. This problem must be resolved if millimeter-sized scaffolds are to be functionally significant.

However, studies of scaffolds have focused mainly on production and fabrication methods, whereas research on diffusion phenomena has received significantly less attention. Therefore, the current study used the fluorescence recovery after photobleaching (FRAP) method to examine microscale diffusion through patterns constructed by electrospinning [29]. Microscale diffusion through an alginate hydrogel was also examined for comparison. The diffusion coefficient of a 2D porous sheet is sometimes measured using donor and receiver chambers divided by the sheet [30]. However, this approach determines only the diffusion coefficient of the entire sheet, which depends on micro structural properties (volume fraction and arrangement of struts, total thickness, uniformity, *etc.*) and properties of strut constituent. The advantage of the FRAP method is that a small region can be assessed, which allows us to measure the diffusion coefficient in the line pattern which are only affected by nanoscale structure rather than the entire patterned mesh. We also simulated cell concentrations in the scaffold to predict the efficiency of a scaffold with permeable struts. The aim of this study was to evaluate and predict the advantages of a scaffold with permeable struts and provide guidelines for designing such a scaffold.

## 2. Results and Discussion

### 2.1. Fabrication of the Electrospun Nanofibrous Patterns

**Figure 1:**
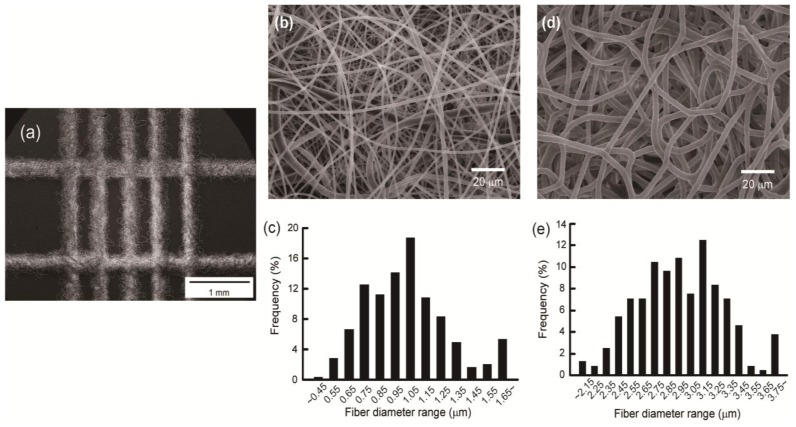
Scanning electron microscopy (SEM) micrographs and fiber diameter distribution charts of the nanofibrous structures. (**a**) Ladder-patterned nanofibrous structure. Scale bar is 1 mm; (**b**,**d**) SEM micrographs of *Q*_p_ = 0.1 mL/h (**b**) and *Q*_p_ = 0.5 mL/h (**d**) samples. Scale bars are 20 μm; (**c**,**e**) Fiber diameter distribution charts of *Q*_p_ = 0.1 mL/h (**c**) and *Q*_p_ = 0.5 mL/h (**e**) samples.

The ladder-patterned nanofibrous structure fabricated using DWES is shown in Figure 1a. A grid-shaped pattern was created using the DWES method, and each line of the pattern was formed by tangled nanofibers. Width and thickness of each line are 309.16 ± 33.56 μm and 78.15 ± 5.12 μm, respectively.

Figure 1b,d present scanning electron micrographs of the electrospun patterns and Figure 1c,e show the nanofiber diameter distribution. To examine differences in the structure of the pattern and diffusion within the electrospun samples according to solution flow rate (*Q*_p_), the scaffold samples were prepared with PCL at flow rates of 0.1 mL/h (Figure 1b,c) and 0.5 mL/h (Figure 1d,e). As shown in Figure 1b–e, the higher the flow rate, the thicker the nanofiber. The average radius and standard deviation of the nanofibers under each process condition were 2.923 ± 0.340 μm at *Q*_p_ = 0.5 mL/h and 1.007 ± 0.381 μm at *Q*_p_ = 0.1 mL/h.

### 2.2. Diffusion Coefficient Measurements Using FRAP

**Figure 2:**
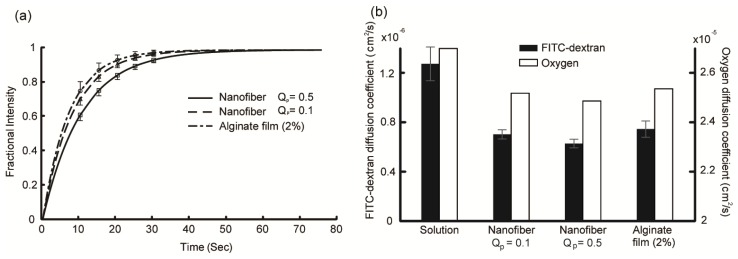
Results of fluorescence recovery after photobleaching (FRAP) measurements. (**a**) Fractional fluorescence intensity recovery curves at flow rates of 0.5 and 0.1 mL/h electrospun line patterns and the 2% calcium alginate hydrogel. Error bars represent the standard deviations; (**b**) Diffusion coefficients of 3-kDa FITC-dextran and oxygen in the electrospun line patterns, 2% calcium alginate hydrogel, and water at 37 °C. Oxygen diffusion coefficients are estimated values from Equation 2 and the diffusion coefficient of 3-kDa FITC-dextran. Error bars represent the standard deviations.

Figure 2a shows the recovery curves of 3-kDa FITC-dextran for the *Q*_p_ = 0.1 and *Q*_p_ = 0.5 line patterns and 2% calcium alginate hydrogel. Five to seven curves obtained from three to five samples were averaged for each curve. The position of the bleaching spot was changed each time to obtain a single recovery curve. As shown in Figure 2a, the recovery rates of the *Q*_p_ = 0.1 mL/h sample and 2% calcium alginate hydrogel were slightly faster than that of the *Q*_p_ = 0.5 mL/h sample. However, the differences were not statistically significant (*p* > 0.05). The diffusion coefficient was calculated from the recovery curve using Equation (1) [31],

(1) $$D=0.224\times \frac{\omega^{2}}{t_{1/2}}$$

where *t*_1/2_ is the half-time of recovery and *ω* is the radius of the bleaching spot. The calculated diffusion coefficients of 3-kDa FITC-dextran in the electrospun line patterns and alginate hydrogel are shown as solid black bars in Figure 2b. The diffusion coefficient in water was also measured and is shown for reference. The measured diffusion coefficient of 3-kDa FITC-dextran in water (1.28 × 10^−6^ cm^2^/s) was reasonably consistent with other reports (1.48 × 10^−6^ cm^2^/s for 4-kDa FITC-dextran [32] and 0.75 × 10^−6^ cm^2^/s for 9.4-kDa FITC-dextran [33]).

The oxygen diffusion coefficient was roughly estimated from the diffusion coefficient of 3-kDa FITC-dextran using Equation (2) [29,34],

(2) $$\frac{D}{D_{0}}=\text{exp}\left(-\sqrt{\varphi_{v}}\frac{r_{s}}{r_{f}}\right)$$

where *D*_0_ is the diffusion coefficient of a test molecule in water, *D* is the diffusion coefficient of a test molecule in the sample, *φ_v_* is the volume fraction of fiber in the sample, *r*_s_ is the radius of the test molecule, and *r*_f_ is the radius of the fiber. This equation describes the effect of barriers formed by randomly distributed long molecular fibers on the diffusion of a molecule [34]. In this equation, $-\sqrt{\emptyset_{v}}/r_{f}$ is a property of the sample and can be calculated using the measured diffusion coefficients of 3-kDa FITC-dextran for each sample. The radius of the 3-kDa FITC-dextran molecule is 1.2 nm [35] and that of oxygen is 0.14 nm [36]. The oxygen diffusion coefficient in 37 °C water is 2.68 × 10^−5^ cm^2^/s [37]. The white bars in Figure 2b indicate the oxygen diffusion coefficient of each sample. The oxygen diffusion coefficient was estimated to be ~92%–94% of that in water for each sample. Equation (2) can overestimate the diffusion coefficient for small molecule [38]; however, the estimated oxygen diffusion coefficient in the alginate hydrogel (2.52 × 10^−5^ cm^2^/s) is in good agreement with that reported previously (2.54 × 10^−5^ cm^2^/s at 30 °C [39]).

### 2.3. Cell Density Distribution Simulated within the Spatially Patterned Scaffold

**Figure 3:**
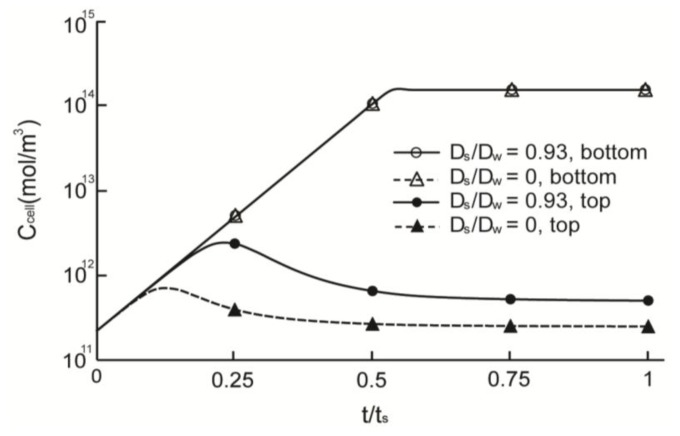
Time based simulation results for the quantity of cells in the various scaffold models. Two points (bottom and top) for electrospun line patterns (*D*_s_/*D*_w_ = 0.93) and for the non-diffusion strut model (*D*_s_/*D*_w_ = 0).

**Figure 4:**
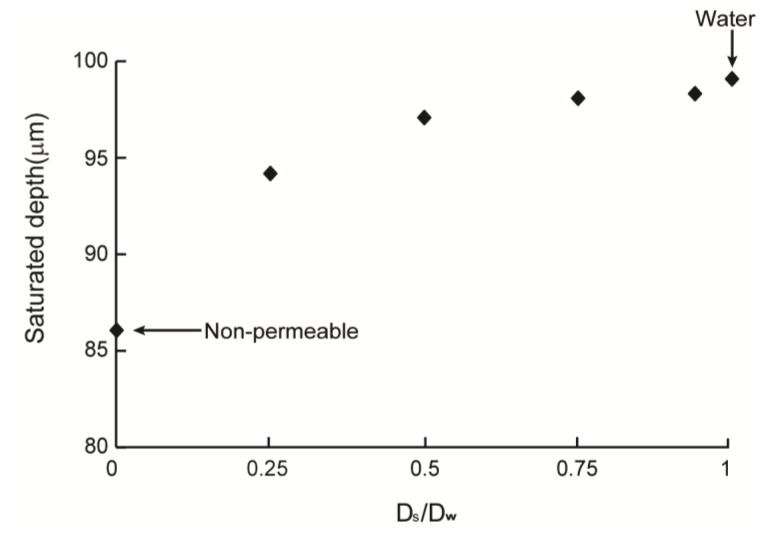
Saturated depth according to strut diffusion coefficient.

Figure 3 illustrates the simulation results of cell concentrations in scaffolds with permeable (*D*_s_/*D*_w_ = 0.93) and non-permeable (*D*_s_/*D*_w_ = 0) struts. The saturation time, *t*_s_, was defined as the time required to attain steady state for the *D*_s_/*D*_w_ = 1 model (~28 days). The cell densities of both permeable and non-permeable cases reached a predetermined maximum value after 0.54 *t*_s_ at the top surface of the scaffold that was directly exposed to fresh media. However, cell density at the bottom surface peaked at 0.13 *t*_s_ and 0.23 *t*_s_ for the non-permeable and permeable strut scaffolds, respectively; moreover, the maximum cell density was much lower than that of the top surface. After peaking, the cell density decreased gradually and reached a steady state. Cell densities were saturated at 98 μm for the permeable strut (*D*_s_/*D*_w_ = 0.93) scaffold and at 86 μm for the non-permeable strut scaffold. Figure 4 shows the saturated depth, which is defined as the depth at which the cell density reaches a predetermined maximum as a function of *D*_s_. The maximum saturated depth was ~99 μm; this value was obtained when the oxygen diffusion coefficient in the strut was identical to that in water, which simulated the situation when the strut region was just filled with water with no obstacles. In the case of the scaffold constructed with a strut diffusion coefficient of 0.75*D*_w_, the saturation depth was similar to the maximum saturated depth, as shown in Figure 4. The oxygen diffusion coefficients of various hydrogels, are 57%–94% [39,40] that of water; therefore, hydrogel strut scaffolds have similar saturated depth when the scaffold strut contains only with water.

**Figure 5:**
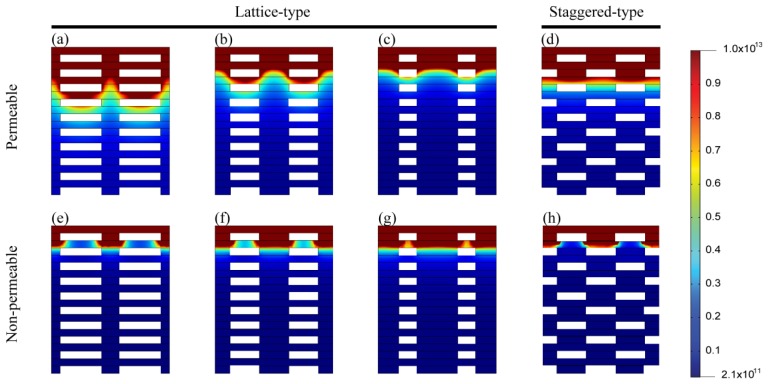
Cell density distribution at *t*_s_. (**a**–**c**) Permeable (*D*_s_ = 0.93 *D*_w_) strut with a lattice-type arrangement. α = 70% (**a**), 50% (**b**) and 30% (**c**); (**d**) Permeable (*D*_s_ = 0.93 *D*_w_) strut with a staggered type arrangement. α = 50%; (**e**–**g**) Non-permeable strut with a lattice-type arrangement. α = 70% (**e**), 50% (**f**) and 30% (**g**); (**h**) Non-permeable strut with a staggered-type arrangement. α = 50%.

Figure 5 shows the cell density distribution according to volume fraction (a–c, e–g) strut arrangement (d and f) in permeable (*D*_s_ = 0.93*D*_w_, a–d) and non-permeable struts (e–h), respectively. In the case of the non-permeable strut scaffold, the cell density distribution was not altered markedly according to the strut arrangement. Additionally, as the volume fraction of the strut (α) increased, the depth to which cells grew to confluence decreased, albeit not to a marked extent.

However, the cell density distribution changed significantly with the strut volume fraction in the case of the permeable strut model. The penetration depth increased as the strut volume fraction increased because the strut region acts as an oxygen-diffusion pass and cell-free region in which oxygen is not consumed. Figure 5b, d show the cell density distribution of scaffolds that had the same volume fraction but different strut arrangements. The cell density was distributed more evenly for the staggered arrangement than for the latticed arrangement, and penetration depth was slightly greater for the staggered arrangement. However, the tendency was the opposite for the non-permeable strut scaffold.

The maximum thicknesses of the scaffolds in this study differed according to the strut width and height and the pore size. However, our analyses demonstrate that the permeable strut scaffold, such as when fabricated using the DWES method, is advantageous for the transfer of materials and for cell growth. We previously reported several preliminary cell culture tests using patterns or scaffolds fabricated by DWES electrospinning [27,41]. The scaffolds fabricated by the DWES method maintained their structures for minimum 14 days, and DWES-patterned scaffolds exhibited superior cell dispersion performance compared with scaffolds fabricated by convectional electrospinning or salt leaching [41].

Numerous studies of scaffolds have been published; however, few have measured the cell density distribution within scaffolds according to their thickness. Therefore, we have to date been unable to verify our simulation results. Nevertheless, Dunn *et al.* [28] cultured MC3T3 pre-osteoblast cells in a 3D scaffold constructed using a solvent casting/particulate-leaching technique with polylactide-co-glycolide. Cells at the edge of the scaffold grew most efficiently; the growth rate decreased gradually with increasing depth; at 1 mm from the edge, growth was negligible. However, these results cannot be compared directly with our findings because of differences in scaffold shapes, materials, and production methods. Also, they did not measure the diffusion coefficient of the scaffold. Androjna *et al.* [30] measured oxygen diffusion coefficient through natural extracellular matrices, such as small-intestine submucosa, human dermis and canine fascia lata. The range for the effective diffusion coefficients was found to be approximately 7 × 10^−6^–4 × 10^−5^ cm^2^/s. They estimated the critical size of cell-seeded scaffold that can be cultured *in vitro*, and those were 0.5–2.0 mm. However, this should be considered to be an initial estimate because the diffusion coefficients are representative of only initial material properties and culture conditions.

## 3. Materials and Methods

### 3.1. DWES Apparatus

**Figure 6:**
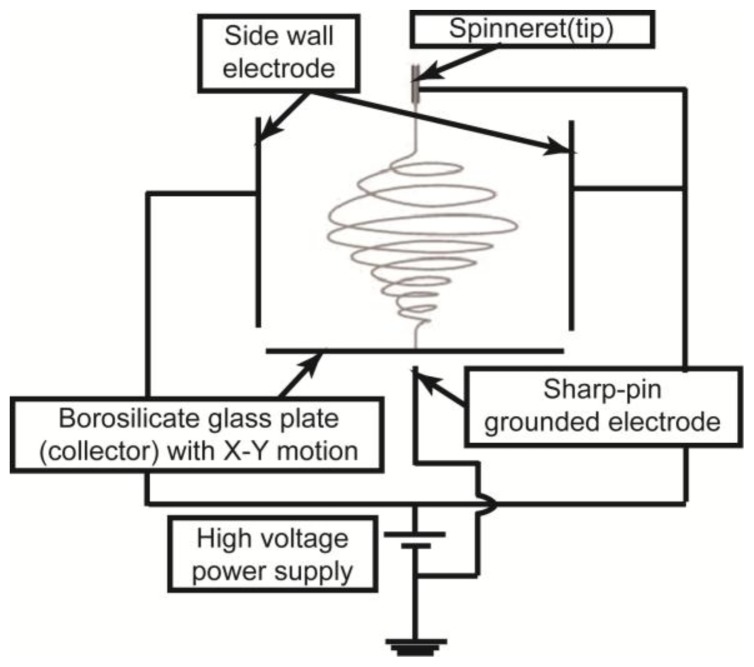
Schematic diagram of the direct-write electrospinning (DWES) apparatus.

Figure 6 presents a schematic diagram of the DWES apparatus used in this study. Complete details regarding the DWES apparatus are described in reference [27]. The DWES apparatus employs the concepts of electric field shaping and a collector scanning system. To shape the electric field, we used a sidewall electrode with an electric potential identical to that of the spinneret, a sharp-pin grounded electrode, and a 27-G needle spinneret. As a result, the electrospun nanofibers were concentrated toward the sharp-pin grounded electrode. A thin borosilicate glass plate with a thickness of 100 μm was placed between the spinneret and the sharp-pin electrode to collect the focused electrospun nanofibers. The glass plate collector had X-Y planar motion as it was placed on an X-Y motion stage. Using this apparatus, a single layer of the nanofibrous pattern was fabricated along the scan path of the collector. We used a layer-by-layer approach to increase the thickness of the nanofibrous pattern.

### 3.2. Sample Preparation

PCL (Sigma, St. Louis, MO, USA) was used for electrospinning. PCL (*MW* = 70,000–90,000) was dissolved in 99.5% pure chloroform (Samchun Pure Chemical Co., Ltd., Pyeongtaek, Korea) to a final concentration of 8.8%. The flow rate of the polymer solution was controlled using a syringe pump. The tip-to-collector distance was ~70 mm, and the voltage applied to the spinneret was 25–30 KV. The scan speed of the collector was 30 mm/s. We used a film structure instead of a line pattern to evaluate the alginate hydrogel. To fabricate a 2% hydrogel film, sodium alginate (Sigma, St. Louis, MO, USA) was dissolved in distilled deionized water at a final concentration of 2 wt%, and calcium carbonate (Junsei Chemical, Tokyo, Japan) was added as a cross-linking agent at a final concentration of 72 mM. G-Glucono-δ-lactone (Sigma, St. Louis, MO, USA) was then added to the solution at a molar ratio of 0.5:1 to maintain pH. The sodium alginate hydrogel was maintained at 37 °C for at least 24 h during cross-linking. The thickness of the fabricated film (~70 μm) was similar to that of the line pattern.

### 3.3. FRAP Method

**Figure 7:**
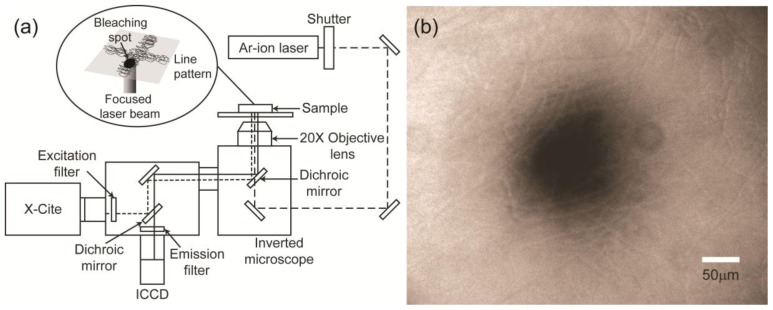
Fluorescence recovery after photobleaching (FRAP) system for diffusion coefficient measurements. (**a**) Experimental setup; (**b**) Image of a bleaching spot.

Figure 7a shows a schematic of FRAP measurement of the diffusion coefficient in the nanofibrous line patterns fabricated by electrospinning and alginate hydrogel film. FRAP is an optical technique that is capable of measuring the diffusion coefficient of fluorescently labeled molecules. It begins with the bleaching of a particular area using a fast and strong light pulse, such as a laser. The diffusion coefficient is calculated by analyzing the recovery curve of the fluorescence intensity at the bleached spot. In this study, a 488-nm Ar-ion laser (Melles Griot, Albuquerque, NM, USA) focused by a 20× objective lens was used to create a bleach spot, and the bleaching time was adjusted to 257 ms using a mechanical shutter. The bleached spot and the recovery process were captured immediately after bleaching using a cooled CCD camera (The Cooke Corp., Romulus, MI, USA) and AQM6 software (Kinetic Imaging, Nottingham, UK). X-cite (Exfo Photonic Solutions Inc., Mississauga, ON, Canada) with an ND filter was used as a light source to observe photobleaching and recovery. The bleaching position was at the center of the line pattern, and we confirmed that the radius of the bleaching spot (~50 μm) was smaller than the width of the pattern (Figure 7b).

We used 3-kDa FITC-dextran (Invitrogen, Carlsbad, CA, USA) as a fluorescent probe. The scaffold was soaked in a FITC-dextran solution (100 mg/mL) under a vacuum for ~5 min to allow the solution to penetrate the pattern. The fluorescence signal was stable prior to bleaching. An inverted microscope (Olympus, Tokyo, Japan) with a 20× objective lens was used to form the bleaching spot and capture recovery.

### 3.4. Simulation of Cell Density Distribution in a Scaffold

**Figure 8:**
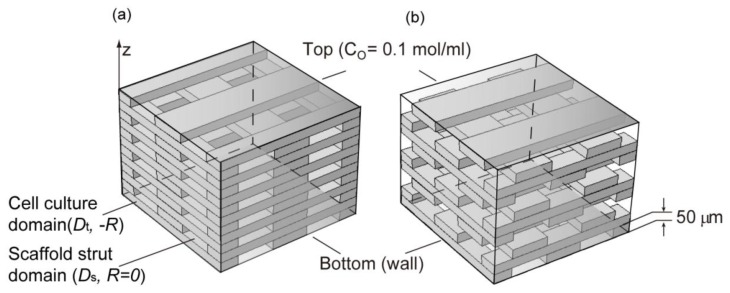
Schematic representation of the simulation domain. (**a**) Lattice model; (**b**) Staggered model.

The simulation was performed using the hypothetical 3D scaffold model shown in Figure 8. The “scaffold strut domain” in Figure 8 simulates the strut of a scaffold with an oxygen diffusion coefficient, *D*_s_. The “cell culture domain”, simulates the space between the struts; cells are assumed to be cultured only in this domain. The height of each strut was set to 50 μm; therefore, the height of each layer of the scaffold was 50 μm. The mesh pattern was created by rotating each layer 90° and stacking layers on top of each other. The total thickness was set to 1000 μm. The diffusion coefficient of the cell culture domain was assumed to be the oxygen diffusion coefficient in typical tissue culture system (*D*_t_) [28]. The diffusion coefficient of the scaffold strut domain *D*_s_ was altered from 0 to 2.68 × 10^−5^ cm^2^/s; that is, the diffusion coefficient of oxygen in water (*D*_w_) [37]. The initial oxygen concentration within the scaffold model was assumed to be the maximum dissolved oxygen concentration (*C*_0_) [28], and the bottom of the scaffold model was assumed to be a wall. The top surface of the scaffold model was assumed to be exposed to fresh media; therefore, the oxygen concentration of the top surface was maintained. Other boundaries were assumed to be symmetrical so that conditions were similar to the culture conditions of a flat, board-shaped scaffold. Oxygen consumption in the cell culture domains was established using the Michaelis-Menten equation (Equation (3)): [42].

(3) $$R=\rho_{cell}\frac{V_{\text{max}}\;C}{K_{m}+C}$$

This equation was derived based on oxygen consumption in spaces within which cells were present. The initial and maximum cell densities were set at 2.1 × 10^11^ cell/m^3^[28] and 1.5 × 10^14^ cell/m^3^[43,44], respectively. The Monod equation (Equation (4)) was applied to simulate cell proliferation.

(4) $$r_{g}=\frac{r_{g,\text{max}}\;C}{K+C}$$

(5) $$r_{g,\text{max}}=\frac{1}{t_{d}}$$

where *r*_g_ is doubling rate, *r*_g,max_ is the maximum cell population doubling rate and *t*_d_ is minimum cell division time [45].

The effect of the strut volume fraction (α = volume of the strut/total scaffold volume) and arrangement were also investigated. Applied strut arrangements were chosen to be of the “lattice type” (Figure 8 left model), which is a criss-cross-shaped truss structure, and the “staggered type” (Figure 8 right model), which is formed by a zigzag shape. The constants in this equation are defined in Table 1, and the analysis was conducted using Comsol Multiphysics version 4.2 (Comsol Inc., Burlington, VT, USA).

## 4. Conclusions

The diffusion characteristics in a spatially patterned scaffold constructed of a permeable strut were investigated. The diffusion coefficients of fluorescent dyes within the DWES line patterns and alginate hydrogel film were measured using FRAP. We observed no significant differences in diffusion coefficients among the alginate hydrogel and DWES line patterns fabricated with polymer supplied at a flow rate of 0.1 and 0.5 mL/h. The oxygen diffusion coefficient was estimated to be ~92%–94% of the diffusion coefficient of water.

A diffusion simulation was performed in the various scaffold models using the estimated oxygen diffusion coefficient. The saturated depths to which cells could grow to confluence were 86 μm for the non-permeable strut scaffold and 99 μm for the permeable strut (*D*_s_/*D*_w_ = 1) scaffold when the strut volume fraction was 50%. Therefore, the permeable strut scaffold could be made ~15% thicker.

We found good diffusion within both the electrospun scaffold and the alginate hydrogel. Therefore, the tissue mass regenerated by bioengineering, which to date has been restricted due to mass transfer of adequate gas and nutrients, might be increased by developing our approach further.

## Figures and Tables

**Table 1:** Parameter values and definitions.

Parameter	Definition	Value	Ref.
*V*_max_	Maximum cellular oxygen consumption rate	3.3 × 10^−16^ mol/cell/s	[46]
*K*_m_	Half-maximum rate oxygen concentration	3.79 × 10^−3^ mol/m^3^	[47]
*K*	Saturation constant in Monod kinetics	3 nmol/mL	[46]
*C*_0_	Maximum dissolved oxygen concentration	0.1 mol/m^3^	[28]
*D*_t_	Oxygen diffusion coefficient in typical tissue culture sysyem	2.0 × 10^−5^ cm^2^/s	[28]
*D*_s_	Oxygen diffusion coefficient in scaffold strut domain	0–2.68 × 10^−5^ cm^2^/s	-
*t*_d_	minimum cell division time	36.5 h	[45]
